# Diagnostic Value of CA 19-9 and Carcinoembryonic Antigen for Pancreatic Cancer: A Meta-Analysis

**DOI:** 10.1155/2018/8704751

**Published:** 2018-11-21

**Authors:** Haibo Xing, Jing Wang, Yanling Wang, Mengting Tong, Hong Hu, Changxin Huang, Da Li

**Affiliations:** ^1^Intensive Care Department, Xiasha Campus, Sir Run Run Shaw Hospital, Zhejiang University School of Medicine, Hangzhou, Zhejiang, China; ^2^Medical Oncology Department, Sir Run Run Shaw Hospital, Zhejiang University School of Medicine, Hangzhou, Zhejiang, China; ^3^Medical Oncology Department, Xiasha Campus, Sir Run Run Shaw Hospital, Zhejiang University School of Medicine, Hangzhou, Zhejiang, China; ^4^Medical Oncology Department, The Affiliated Hospital of Hangzhou Normal University, Hangzhou, Zhejiang, China

## Abstract

**Background:**

CA 19-9 and carcinoembryonic antigen (CEA) are widely used for the diagnosis of pancreatic cancer. The purpose of the present study was to compare the diagnostic value of CA 19-9 with CEA for pancreatic cancer.

**Methods:**

The studies were obtained from electronic searches conducted in PubMed, Embase, and Cochrane Library databases until December 2017. The keywords included diagnosis of pancreatic cancer, CA 19-9, and CEA. The ratio of sensitivity, the specificity, the diagnostic odds ratio (DOR), and the summary of the receiver operating characteristic (SROC) with regard to CA 19-9 and CEA were measured using the random effects model. The current study included 13 studies that comprised 4,537 participants and 1,277 patients with pancreatic cancer.

**Results:**

The levels of CA 19-9 for use for the detection of pancreatic cancer were associated with higher sensitivity (ratio of sensitivity: 1.54; 1.31–1.81; *P* < 0.001), DOR (DOR: 3.50; 95% CI: 2.24–5.45; *P* < 0.001), and AUC (ratio of AUC: 1.24; 95% CI: 1.15–1.33; *P* < 0.001) compared with the variable CEA, while no significant difference between CA 19-9 and CEA was noted with regard to specificity (ratio of specificity: 0.97; 95% CI: 0.89–1.06; *P* = 0.517). The findings of the subgroup analyses suggested that different cutoff values of CA 19-9 and CEA might affect the diagnostic value.

**Conclusions:**

The findings of the present study indicated that CA 19-9 levels were associated with higher sensitivity, DOR, and AUC compared with the corresponding levels of CEA with regard to the diagnosis of pancreatic cancer.

## 1. Introduction

Pancreatic cancer is the fourth leading cause of cancer-related mortality for both men and women worldwide, and an estimated 338,000 new cases are diagnosed every year [1]. Pancreatic cancer is a highly lethal disease, which is characterized by a difficult diagnosis and an aggressive progression [2]. The majority of the patients with pancreatic cancer do not survive within 2 years of the diagnosis, whereas the 5-year survival rate is less than 5.5% [3–6]. Consequently, accurate diagnosis at an early stage is a significant determinant that is required to improve the prognosis of pancreatic cancer.

Despite advanced imaging and invasive endoscopic approaches that are widely used to differentiate pancreatic carcinoma from benign pancreatic disease, the early diagnosis of pancreatic cancer remains a significant challenge [7, 8]. Several serum tumor markers have been used effectively as a noninvasive diagnostic approach for the early detection of pancreatic cancer. However, some of them are not sufficiently sensitive and/or specific to distinguish between the benign and malignant forms of the disease. Huang and Liu conducted a meta-analysis and concluded that serum CA 19-9 plays an important role in the diagnosis of pancreatic cancer [9]. The pooled sensitivity and specificity were 0.80 (0.77–0.82), whereas the diagnostic odds ratio (DOR) and the area under the curve (AUC) were 14.79 (8.55–25.59) and 0.87, respectively [9]. Furthermore, the tumor marker, carcinoembryonic antigen (CEA), has been investigated for the diagnosis of several cancers and it was proposed as a promising biomarker [10–14]. However, the diagnostic value between CA 19-9 and CEA for the detection of pancreatic cancer remains controversial.

Several studies have investigated the diagnostic value of CA 19-9 and CEA for pancreatic cancer and reported inconsistent results. Furthermore, whether CA 19-9 is superior to CEA for the detection of pancreatic cancer remains controversial and a direct comparison of these two markers has not been examined to date. We therefore conducted a meta-analysis in order to evaluate the diagnostic value of CA 19-9 and CEA for the diagnosis of pancreatic cancer. In addition, the parameters sensitivity, specificity, DOR and AUC were compared between the two tumor biomarkers.

## 2. Materials and Methods

### 2.1. Data Sources, Search Strategy, and Selection Criteria

The present study was conducted in accordance with the guidelines for the Preferred Reporting Items for Systematic Reviews and Meta-Analyses (PRISMA) [15]. We performed electronic searches in PubMed, Embase, and Cochrane Library from December 2016 in order to identify studies that used CA 19-9 and CEA in the diagnosis of pancreatic cancer. The following search terms were used: “CA-19-9 Antigen” and “Carcinoembryonic Antigen” and “Pancreatic Cancer.” The details of the searching strategies in each electronic database are listed in the Supplemental file. The additional publications in the reference list and the citation sections of the recovered articles were also searched. Letters, abstracts, and conference proceedings were excluded due to certain discrepancies. Notably, inconsistencies were common between the results published in meeting abstracts and those published in the journal articles. The publication languages were limited to English.

The literature search was independently undertaken by 2 authors using a standardized approach. Any inconsistencies between these 2 authors were settled by the primary author until a consensus was reached. The study was eligible for inclusion if the following criteria were met: (1) the study should have sufficient data to calculate the true positive (TP), false positive (FP), false negative (FN), and true negative (TN) values of CA 19-9 and CEA for the diagnosis of pancreatic cancer; (2) the study was designed to provide a direct comparison of CA 19-9 and CEA; and (3) all patients were required to have a histological diagnosis of pancreatic cancer. The articles that did not include raw data including reviews, case reports, comments, editorials, and letters were excluded.

### 2.2. Data Collection and Quality Assessment

A total of 2 authors reviewed the abstract first independently and then summarized the selected studies. The inconsistencies that were present were settled by group discussion until a consensus was reached. The relevant data abstracted were listed as follows: first author, publication years, country, sample size, mean age, number of male and female, golden standard, cases of pancreatic cancer, TP, FP, FN, and TN. The Quality Assessment of Diagnostic Accuracy Studies (QUADAS) tool [16, 17] was used to evaluate the quality of the studies included in this meta-analysis independently by the two authors. Each of the assessment included 7 items and a response as “yes,” “no,” and/or “unclear”. The answer of “yes” indicated that a study's risk bias could be judged as low, while “no” and “unclear” indicated that the risk of bias could be judged as high.

### 2.3. Statistical Analysis

The summary sensitivity, specificity, diagnostic odds ratio (DOR), area under the curve (AUC), and corresponding 95% confidence intervals (CIs) were calculated from TP, FP, FN, and TN, which were extracted from each study prior to data pooling. The bivariate random effects [18] were applied in order to analyze sensitivity, specificity, and DOR, and the hierarchical regression model was used to analyze receiver operating characteristic (SROC) curve and the AUC [19]. The ratio of sensitivity, specificity, DOR, and AUC were calculated by the random effects model [20]. *Q* statistics and *I*-squared test were used to estimate the heterogeneity of each individual study that contributed to the pooled estimate. A *P* value higher than 0.1 (*P* > 0.10) indicated no significant heterogeneity, while a *P* value lower than and/or equal to 0.1 (*P* ≤ 0.10) indicated significant heterogeneity for the *Q* statistical analysis [21, 22]. Subgroup analyses were further conducted for sensitivity, specificity, and DOR based on the cutoff values of CA 19-9 and CEA. The visual inspections of the funnel plots were constructed by Deeks' asymmetry test for CA 19-9 and CEA [23]. All statistical analyses were conducted using the Stata software (version 10.0; Stata Corporation, TX, USA).

## 3. Results

### 3.1. Study Selection Process

**Figure 1:**
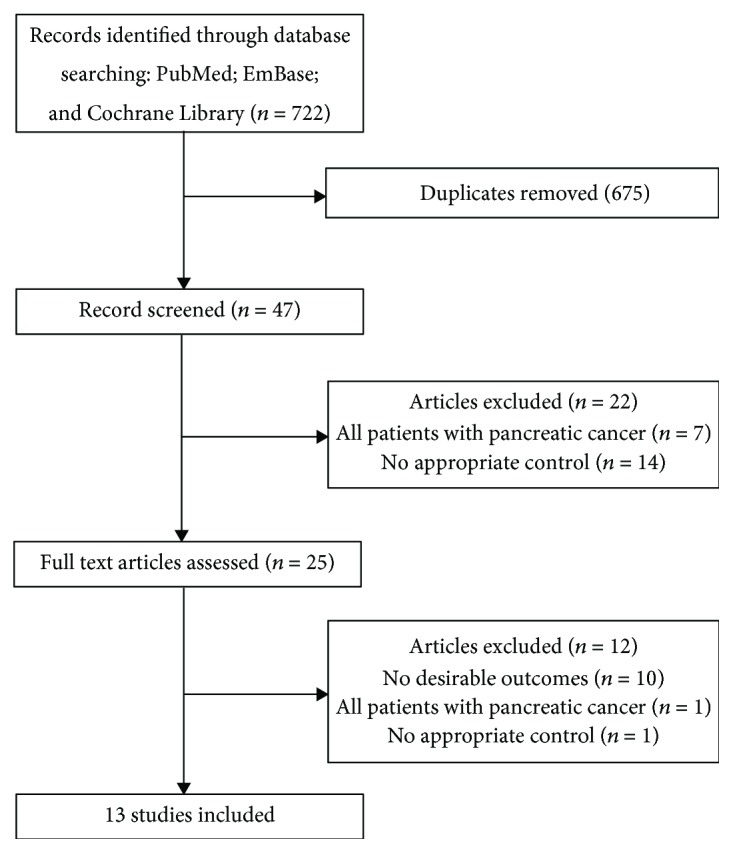
Flow diagram of the literature search and the study selection process.

The study selection process is presented in Figure 1. Based on the initial electronic searches, 722 potential articles were identified and 675 were excluded following a preliminary review of titles and abstracts. Following a detailed evaluation of 47 potentially eligible studies, 13 studies were included comprising 4,537 participants and 1,277 patients diagnosed with pancreatic cancer [24–36]. A manual search of the reference lists from relevant studies did not yield any new additional eligible studies.  Table 1 summarizes the general characteristics of the included studies.

### 3.2. Study Characteristics

The sample size ranged from 41 to 2,522, while the range of the pancreatic cancer cases was 10 to 641 from the 13 selected studies. A total of 7 studies were conducted in Europe, and the remaining 6 studies were conducted in Asia. A total of 11 and 2 studies used the levels of 37 U/ml and 35 U/ml, respectively, as cutoff values for the biomarker CA 19-9. Furthermore, 10 studies used 5 ng/ml in CEA as a cutoff value, for the biomarker CEA. The remaining 3 studies used 8.4, 2.5, and 3.0 ng/ml, respectively, as cutoff values for CEA. The QUADAS quality assessment of the individual study is presented in the Supplemental file (Table S1).

### 3.3. Sensitivity

**Figure 2:**
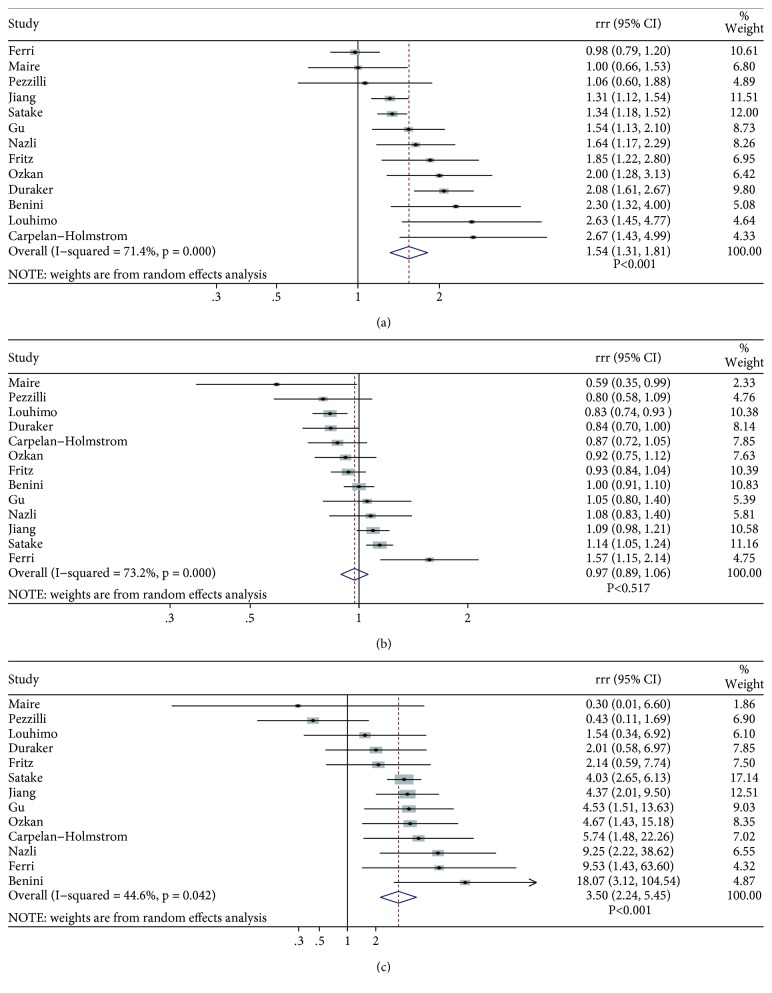
The sensitivity (a), specificity (b), and DOR (c) between CA 19-9 and CEA for the diagnosis of pancreatic cancer.

The summary sensitivity values for CA 19-9 and CEA were 0.80 (0.72–0.86; *P* < 0.001) and 0.50 (0.40–0.59; *P* < 0.001), respectively (Supplemental file: Figures S1-S2). We noted that CA 19-9 was associated with higher sensitivity than CEA (ratio of sensitivity: 1.54; 95% CI: 1.31–1.81; *P* < 0.001; Figure 2(a)) and substantial heterogeneity was observed (*I* ^2^ = 71.4%; *P* < 0.001). The findings of the sensitivity analysis indicated that the conclusions were not affected following sequential exclusion of each study. The findings of the subgroup analyses were consistent with those noted in the overall analysis (Table 2). However, we noted higher sensitivity for CA 19-9 compared with CEA when the levels of 37 U/ml were used as a cutoff value as opposed to the levels of 35 U/ml (ratio between subgroup: 0.55; 95% CI: 0.35–0.87; *P* = 0.011).

### 3.4. Specificity

The summary specificity values of CA 19-9 and CEA were 0.75 (0.68–0.80; *P* < 0.001) and 0.78 (0.70–0.85; *P* < 0.001), respectively (Supplemental file: Figures S1-S2). There were no significant differences noted between CA 19-9 and CEA with regard to specificity (ratio of specificity: 0.97; 95% CI: 0.89–1.06; *P* = 0.517; Figure 2(b)). Although substantial heterogeneity was observed (*I* ^2^ = 73.2%; *P* < 0.001), the sensitivity analysis demonstrated that the results were not affected by the sequential exclusion of the individual studies. Subgroup analysis indicated that CA 19-9 was associated with lower specificity compared with CEA, when 35 U/ml was used as a cutoff value (ratio of specificity: 0.84; 95% CI: 0.76–0.93; *P* < 0.001), whereas no other significant differences for the variable specificity were observed (Table 2). Moreover, when the cutoff value of CA 19-9 was at 37 U/ml, a higher specificity was noted compared with that at 35 U/ml as the cutoff value used for CEA (ratio between subgroups: 1.19; 95% CI: 1.04–1.36; *P* = 0.011).

### 3.5. DOR

The summary values of the variable DOR for CA 19-9 and CEA were 11.83 (7.43–18.83; *P* < 0.001) and 3.49 (2.44–5.00; *P* < 0.001), respectively (Supplemental file: Figures S3-S4). We noted that when CA 19-9 was used for the diagnosis of the disease, pancreatic cancer was associated with higher DOR than CEA (DOR: 3.50; 95% CI: 2.24–5.45; *P* < 0.001; Figure 2(c)). Although moderate heterogeneity was observed (*I* ^2^ = 44.6%; *P* = 0.042) following sequential exclusion of each study, the conclusions were not altered. Subgroup analyses suggested no significant differences between CA 19-9 and CEA with regard to DOR when 35 U/ml was used as a CA 19-9 cutoff value (DOR: 3.10; 95% CI: 0.85–11.25; *P* = 0.086). Similar conclusions were drawn when the three cutoff values, namely, 2.5, 3.0, and 8.4 ng/ml, were used for CEA (DOR: 3.00; 95% CI: 0.56–16.21; *P* = 0.202) (Table 2).

### 3.6. ROC

**Figure 3:**
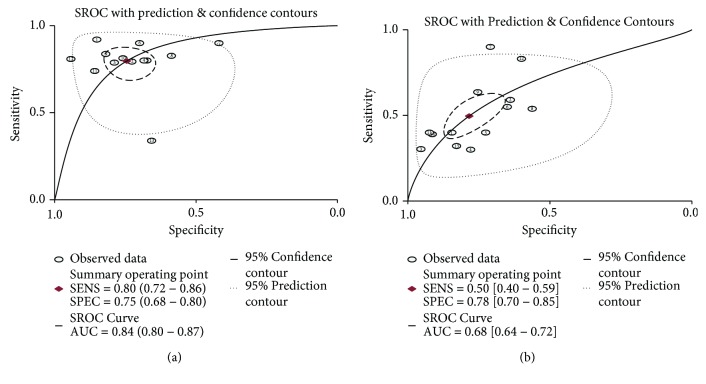
The SROC of CA 19-9 and CEA for the diagnosis of pancreatic cancer.

The ROC curves for the parameter AUC with regard to CA 19-9 and CEA were 0.84 (0.80–0.87) and 0.68 (0.64–0.72), respectively (Figure 3). Furthermore, we noted that CA 19-9 was associated with higher AUC than CEA (ratio of AUC: 1.24; 95% CI: 1.15–1.33; *P* < 0.001).

### 3.7. Publication Bias

**Figure 4:**
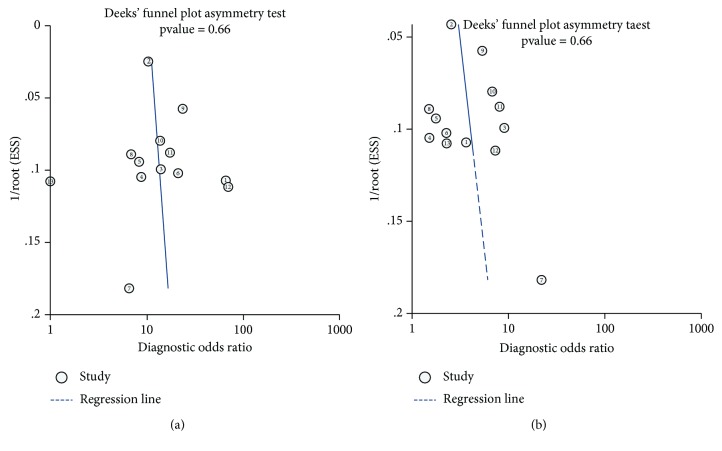
Publication bias for the biomarkers CA 19-9 and CEA.

The review of Deeks' funnel plots could not exclude the potential publication bias for the biomarkers CA 19-9 and CEA (Figure 4). The *P* value for Deeks' funnel plot asymmetry test indicated no evidence of publication bias for CA 19-9 (*P* = 0.66) and CEA (*P* = 0.45).

## 4. Discussion

The current meta-analysis was conducted in order to compare the diagnostic value of CA 19-9 and CEA for the detection of pancreatic cancer. The present comparison study included 4,537 participants and 1,277 cases of pancreatic cancer that were derived from 13 studies with a broad range of characteristics. The findings from the present study suggest that CA 19-9 can be used as a marker with higher sensitivity, specificity, DOR, and AUC compared with CEA that was associated with lower sensitivity, DOR, and AUC. In addition, we noted that CA 19-9 was associated with higher sensitivity, DOR, and AUC for the early detection of pancreatic cancer compared with CEA, whereas no significant difference was noted with regard to specificity. Subgroup analyses indicated that different cutoff values for CA 19-9 and/or CEA might affect the diagnostic value for pancreatic cancer.

A previous meta-analysis suggested that serum CA 19-9 contributed significantly in the detection of pancreatic cancer [9]. Furthermore, Su et al. suggested that the summary diagnostic values of the parameters used for CA 19-9 that were required to differentiate pancreatic cancer from chronic pancreatitis were as follows: sensitivity, 0.81; specificity, 0.81; positive likelihood ratio, 4.08; negative likelihood ratio, 0.24; DOR, 19.31; and AUC, 0.88 [37]. Cao et al. suggested that serum CA 19-9 exhibited a satisfactory specificity (0.88) and a poor sensitivity (0.47) for discriminating benign from malignant pancreatic cystic neoplasms, when the data were pooled [38]. The inherent limitation of this meta-analysis was that the diagnostic value between CA 19-9 and CEA was not examined. The same disadvantage was noted in the specific subpopulation analysis. Consequently, the present meta-analysis was conducted in order to compare the diagnostic value of CA 19-9 with the corresponding diagnostic value of CEA with regard to the direct diagnosis of pancreatic cancer.

The majority of the studies suggested that CA 19-9 exhibited higher sensitivity for the diagnosis of pancreatic cancer compared with CEA, while several studies included reported inconsistent results. Ferri et al. [35] demonstrated that the sensitivity of CA 19-9 for the detection of pancreatic cancer was 0.81 (0.67–0.91), whereas that of CEA was 0.83 (0.69–0.92). Maire et al. [30] indicated that both CA 19-9 and CEA exhibited the same values of sensitivity (0.90) for the diagnosis of pancreatic cancer. Pezzilli et al. [36] suggested that CA 19-9 and CEA exhibited lower sensitivity and reported mean values of 0.34 (0.22–0.48) and 0.32 (0.20–0.46), respectively. The possible reasons for these findings could be attributed to the small sample size of patients used in these studies. In addition, the patient status was different compared with that noted in other studies. Furthermore, the lower cutoff value of CEA was associated with higher sensitivity, whereas the difference between CA 19-9 and CEA required a large sample size in order to ensure sufficient power.

No significant difference was noted for the comparison of the specificity between CA 19-9 and CEA with regard to the detection of pancreatic cancer. Although the majority of the included studies reported consistent results, Maire et al. [30] demonstrated that the specificity of CA 19-9 was 0.42, while that of CEA was 0.71. The authors concluded that CEA exhibited optimal specificity for the preoperative differential diagnosis of benign and malignant intraductal papillary mucinous neoplasms. All of the included patients with intraductal papillary mucinous neoplasms may have affected the specificity of CA 19-9 and thus contributed to this significant difference. Moreover, Louhimo et al. [26] indicated that the specificity of CA 19-9 was 0.79, while that of CEA was 0.95. This discrepancy may be due to the cutoff value of CA 19-9 (35 U/ml), which was lower than the conventional reference value and resulted in higher sensitivity and lower specificity. Conversely, Satake and Takeuchi [25] suggested that the specificity of CA 19-9 was 0.73, while that of CEA was 0.64. The cutoff value of CEA was 2.5 ng/ml and contributed to a lower specificity. Ferri et al. [35] reported a similar conclusion for the differentiation of pancreatic cancer from chronic pancreatitis, whereas the disease status of the participants may affect the specificity when CEA is used to diagnose pancreatic cancer.

The finding of the current study demonstrated that CA 19-9 exhibited higher DOR compared with CEA. A total of 2 studies suggested no significant difference between CA 19-9 and CEA and revealed that the DOR of CA 19-9 was lower than that of CEA for the diagnosis of pancreatic cancer [30, 36]. Furthermore, 3 of the included studies suggested that CA 19-9 exhibited higher DOR, although it was not associated with a statistically significant difference compared with CEA [26, 33, 34]. The value of DOR in each study revealed a positive likelihood ratio and a negative likelihood ratio, and the variation of these two variables might contribute to the aforementioned nonsignificant difference. In addition, CA 19-9 was associated with higher AUC than CEA for the detection of pancreatic cancer (ratio of AUC: 1.24; 95% CI: 1.15–1.33; *P* < 0.001). This finding was consistent with the data derived for the sensitivity and DOR. Finally, the findings of the subgroup analysis were affected by the cutoff values of CA 19-9 and CEA, as expected.

Although the summary of the diagnostic value of CA 19-9 and CEA for the detection of pancreatic cancer was mild, it was unable to provide the necessary information for the diagnosis of pancreatic cancer. These two markers should be recommended for early diagnosis of pancreatic cancer due to convenient, efficient, and noninvasive applications. Furthermore, individuals with higher levels of CA 19-9 and CEA may correlate with higher cancer risk. The present study is the first meta-analysis that directly compares the diagnostic value of CA 19-9 with CEA with regard to the early diagnosis of pancreatic cancer. The cutoff values can be used in order to distinguish the more useful marker and avoid excessive medical examination.

The present study exhibits certain advantages: (1) the study compared the diagnostic value of CA 19-9 and CEA for the detection of pancreatic cancer directly; (2) the ratios of sensitivity, specificity, DOR, and AUC were investigated in order to allow a direct comparison of the diagnostic value of CA 19-9 and CEA; (3) the diagnostic value of CA 19-9 and CEA, based on different cutoff values, was further explored; and (4) the large sample size allowed us to quantitatively evaluate the diagnostic value of CA 19-9 and CEA. Consequently, the findings of this study are robust compared with an individual study.

The limitations of this study should be highlighted as follows: firstly, the characteristics of the participants were different across the included studies and these factors might have affected the diagnostic value of CA 19-9 and CEA; secondly, the stratified analyses based on the characteristics of patients including jaundice, blood groups were not conducted due to the data that were not available; thirdly, the sources of heterogeneity among the included studies were not completed due to the rare reporting of the characteristics of the studies and patients; and finally, in a meta-analysis of published studies, publication bias is an inevitable problem.

## 5. Conclusions

The findings of the present study suggest that CA 19-9 exhibits higher sensitivity, DOR, and AUC compared with CEA for the diagnosis of pancreatic cancer, while no significant differences were noted for the parameter specificity. Furthermore, different cutoff values for CA 19-9 and CEA had affected the diagnostic value. Future studies should combine CA 19-9 and CEA with other biomarkers in order to enhance the diagnostic value for the diagnosis of pancreatic cancer and the diagnostic value of these two markers for the distinction of different stages of pancreatic cancer.

## Figures and Tables

**Table 1:** Characteristics of the studies included in the meta-analysis.

Author	Year	Country	*N*	Mean age	Male/female	Gold standard	Cases of pancreatic cancer	Cutoff value of CA 19-9	Cutoff value of CEA
Benini	1988	Italy	193	53.1	117/76	Histology	25	37 U/ml	8.4 ng/ml
Satake	1994	Japan	2522	NA	NA	Pathological	641	37 U/ml	2.5 ng/ml
Louhimo	2002	Finland	320	59.9	189/131	Histology	33	35 U/ml	5 ng/ml
Carpelan-Holmstrom	2002	Finland	286	NA	NA	Histology	30	35 U/ml	5 ng/ml
Ozkan	2003	Turkey	135	57.3	NA	Histopathologic	40	37 U/ml	5 ng/ml
Nazli	2000	Turkey	100	57.1	49/51	Histopathologic	40	37 U/ml	5 ng/ml
Maire	2008	France	41	64.0	14/27	Pathological	10	37 U/ml	5 ng/ml
Gu	2015	China	132	56.5	68/64	Pathological	52	37 U/ml	5 ng/ml
Jiang	2004	China	312	NA	NA	Pathological	129	37 U/ml	5 ng/ml
Duraker	2007	Turkey	181	NA	NA	Histology	123	37 U/ml	5 ng/ml
Fritz	2011	Germany	142	31–87	82/60	Histology	50	37 U/ml	2.5 or 5 ng/ml
Ferri	2016	Spain	82	61.6	48/34	Histopathologic	47	37 U/ml	5 ng/ml
Pezzilli	2016	Italy	91	61.6	54/37	Histology	56	37 U/ml	3 ng/ml

**Table 2:** Subgroup based on different cutoff values of CA 19-9 and CEA.

Outcomes	Group	Ratio of indices and 95% CI	*P* value	Heterogeneity (%)	*P* value for heterogeneity	Ratio between subgroups
Sensitivity						
Cutoff value of CA 19-9	37 U/ml	1.46 (1.25–1.70)	<0.001	70.1	<0.001	0.55 (0.35–0.87)/0.011
	Others	2.65 (1.72–4.08)	<0.001	0.0	0.977	
Cutoff value of CEA	5 ng/ml	1.59 (1.29–1.96)	<0.001	75.9	<0.001	1.10 (0.74–1.64)/0.624
	Others	1.44 (1.03–2.02)	0.034	52.9	0.120	

Specificity						
Cutoff value of CA 19-9	37 U/ml	1.00 (0.92–1.10)	0.945	67.5	0.001	1.19 (1.04–1.36)/0.011
	Others	0.84 (0.76–0.93)	<0.001	0.0	0.673	
Cutoff value of CEA	5 ng/ml	0.96 (0.86–1.06)	0.412	69.8	<0.001	0.94 (0.79–1.12)/0.498
	Others	1.02 (0.89–1.18)	0.757	73.1	0.024	

DOR						
Cutoff value of CA 19-9	37 U/ml	3.55 (2.16–5.83)	<0.001	49.8	0.030	1.15 (0.29–4.57)/0.848
	Others	3.10 (0.85–11.25)	0.086	38.6	0.202	
Cutoff value of CEA	5 ng/ml	3.81 (2.56–5.67)	<0.001	0.0	0.451	1.27 (0.23–7.16)/0.786
	Others	3.00 (0.56–16.21)	0.202	84.3	0.002	

## Supplementary Materials

Supplementary Materials Table S1: quality evaluation of the included studies using the QUADAS tool. Figure S1: the summary of the results with regard to the sensitivity and specificity for CA 19-9. Figure S2: the summary of the results with regard to the sensitivity and specificity for CEA. Figure S3: the summary of the results with regard to the DOR for CA 19-9. Figure S4: the summary of the results with regard to the DOR for CEA.
