# Supplementary material for: Diagnostic Value of CA 19-9 and Carcinoembryonic Antigen for Pancreatic Cancer: A Meta-Analysis
Source: Gastroenterol Res Pract. 2018 Nov 21;2018:8704751. doi: 10.1155/2018/8704751 (PMC6280291; doi:10.1155/2018/8704751)
Supplement: Supplementary Materials — Table S1: quality evaluation of the included studies using the QUADAS tool. Figure S1: the summary of the results with regard to the sensitivity and specificity for CA 19-9. Figure S2: the summary of the results with regard to the sensitivity and specificity for CEA. Figure S3: the summary of the results with regard to the DOR for CA 19-9. Figure S4: the summary of the results with regard to the DOR for CEA. [file 8704751.f1.pdf]

The search strategy in PubMed:

#1 "CA-19-9 Antigen"[Mesh] or “CA-19-9” OR “CA-19-9 Antigen” OR “Antigen, CA-19-9” OR “CA 19 9 Antigen” OR “CA 19.9 Antigen” OR “Antigen, CA 19.9” OR “Gastrointestinal Cancer Antigen” OR “Cancer Antigen, Gastrointestinal” OR “CA 19-9 Antigen” OR “Antigen, CA 19-9” OR “Antigen CA-19-9” or “Antigen CA 19 9” or “CA-19-9, Antigen”

#2 "Pancreatic Neoplasms/diagnosis"[Mesh] or "Pancreatic Neoplasms" or “Neoplasm, Pancreatic" or “Pancreatic Neoplasm" or “Pancreas Neoplasms" or “Neoplasm, Pancreas" or “Neoplasms, Pancreas" or “Pancreas Neoplasm" or “Neoplasms, Pancreatic" or “Cancer of Pancreas" or “Pancreas Cancers" or “Pancreas Cancer" or “Cancer, Pancreas" or “Cancers, Pancreas" or “Pancreatic Cancer" or “Cancer, Pancreatic" or “Cancers, Pancreatic" or “Pancreatic Cancers" or “Cancer of the Pancreas"

#3 "Carcinoembryonic Antigen"[Mesh] OR "Antigen, Carcinoembryonic" OR "CD66e Antigen" OR "Antigens, CD66e" OR "CD66e Antigens" OR "Antigen, CD66e"

#4 ((#1 OR #3) and #2)   Filters: Clinical trial; Humans 175

The search strategy in EMBASE:

'pancreatic neoplasms'/exp OR 'pancreas neoplasia' OR 'pancreas neoplasm' OR 'pancreas tumor' OR 'pancreas tumour' OR 'pancreatic neoplasm' OR 'pancreatic neoplasms' OR 'pancreatic tumor' OR 'pancreatic tumour' OR 'pancreatic cancer'/exp

OR 'pancreas cancer' OR 'pancreatic cancer' AND ('ca 19 9 antigen'/exp OR 'ca 19 9 antigen' OR 'ca 19-9 antigen' OR 'antigen 19-9' OR 'antigen ca 19-9' OR 'ca 19 9' OR 'ca 19-9' OR 'ca-19-9 antigen' OR 'cancer associated antigen 19-9' OR 'cancer associated glycolipid antigen 19-9' OR 'carbohydrate antigen 19-9' OR 'carcinoembryonic antigen'/exp OR 'cea' OR 'antigen, carcinoembryonic' OR 'carcinoembryonal antigen' OR 'carcinoembryonic antigen' OR 'carcinoembryonic protein' OR 'ce antigen' OR 'protein, carcinoembryonic') AND [embase]/lim NOT [medline]/lim AND ('clinical article'/de OR 'clinical study'/de OR 'clinical trial'/de OR 'cohort analysis'/de OR 'controlled study'/de OR 'diagnostic test accuracy study'/de OR 'major clinical study'/de OR 'prospective study'/de OR 'retrospective study'/de) AND [humans]/lim 481

The search strategy in Cochrane library

#1 MeSH descriptor: [CA-19-9 Antigen] explode all trees

#2 “CA-19-9” OR “CA-19-9 Antigen” OR “Antigen, CA-19-9” OR “CA 19 9 Antigen” OR “CA 19.9 Antigen” OR “Antigen, CA 19.9” OR “Gastrointestinal Cancer Antigen” OR “Cancer Antigen, Gastrointestinal” OR “CA 19-9 Antigen” OR “Antigen, CA 19-9” OR “Antigen CA-19-9” or “Antigen CA 19 9” or “CA-19-9, Antigen” or “ca 19 9 antigen” OR “ca 19-9 antigen” OR “antigen 19-9” OR “antigen ca 19-9” OR “ca 19 9” OR “ca 19-9” OR “ca-19-9 antigen” OR “cancer associated antigen 19-9” OR “cancer associated glycolipid antigen 19-9”

#3 MeSH descriptor: [Carcinoembryonic Antigen] explode all trees

#4 "Antigen, Carcinoembryonic" OR "CD66e Antigen" OR "Antigens, CD66e" OR "CD66e Antigens" OR "Antigen, CD66e" or “cea” OR “antigen, carcinoembryonic” OR “carcinoembryonal antigen” OR “carcinoembryonic antigen” OR “carcinoembryonic protein” OR “ce antigen” OR “protein, carcinoembryonic”

#5 MeSH descriptor: [Pancreatic Neoplasms] explode all trees

#6 "Pancreatic Neoplasms" or “Neoplasm, Pancreatic" or “Pancreatic Neoplasm" or “Pancreas Neoplasms" or “Neoplasm, Pancreas" or “Neoplasms, Pancreas" or “Pancreas Neoplasm" or “Neoplasms, Pancreatic" or “Cancer of Pancreas" or “Pancreas Cancers" or “Pancreas Cancer" or “Cancer, Pancreas" or “Cancers, Pancreas" or “Pancreatic Cancer" or “Cancer, Pancreatic" or “Cancers, Pancreatic" or “Pancreatic Cancers" or “Cancer of the Pancreas" or 'pancreas neoplasia' OR 'pancreas tumor' OR 'pancreas tumour' OR 'pancreatic tumor' OR 'pancreatic tumour'

#7 (#1 or #2 or #3 or #4) and (#5 or #6) in trials 66

Table S1. Quality evaluation of the included studies using the QUADAS tool

Study	Question about study design characteristic													
	Representative patient spectrum	Reporting of selection criteria	Reference standard	Absence of disease progression bias	Absence of partial verification bias	Absence of differential verification bias	Absence of incorporation bias	Description of index text execution	Description of reference standard execution	Reference standard blinded	Index test blinded	Absence of clinical review bias	Reporting of uninterpretable/intermediate results	Withdrawal
Benini	Yes	Yes	Yes	Yes	Yes	Yes	Yes	Yes	Yes	Yes	Yes	Yes	No	Yes
Satake	Yes	Yes	Yes	Yes	Yes	Yes	Yes	Yes	Yes	Yes	Yes	Yes	No	Yes
Louhimo	Yes	Yes	Yes	Yes	Yes	Yes	Yes	Yes	Yes	Yes	Yes	Yes	No	Yes
Carpelan	Yes	Yes	Yes	Yes	Yes	Yes	Yes	Yes	Yes	Yes	Yes	Yes	No	Yes

-Holmstr<br>om														
Ozkan	Yes	Yes	Yes	Yes	Yes	Yes	Yes	Yes	Yes	Yes	Yes	Yes	No	Yes
Nazli	Yes	Yes	Yes	Yes	Yes	Yes	Yes	Yes	Yes	Yes	Yes	Yes	No	Yes
Maire	Yes	Yes	Yes	Yes	Yes	Yes	Yes	Yes	Yes	Yes	Yes	Yes	No	Yes
Gu	Yes	Yes	Yes	Yes	Yes	Yes	Yes	Yes	Yes	Yes	Yes	Yes	No	Yes
Jiang	Yes	Yes	Yes	Yes	Yes	Yes	Yes	Yes	Yes	Yes	Yes	Yes	No	Yes
Duraker	Yes	Yes	Yes	Yes	Yes	Yes	Yes	Yes	Yes	Yes	Yes	Yes	No	Yes
Fritz	Yes	Yes	Yes	Yes	Yes	Yes	Yes	Yes	Yes	Yes	Yes	Yes	No	Yes
Ferri	Yes	Yes	Yes	Yes	Yes	Yes	Yes	Yes	Yes	Yes	Yes	Yes	No	Yes
Pezzilli	Yes	Yes	Yes	Yes	Yes	Yes	Yes	Yes	Yes	Yes	Yes	Yes	No	Yes

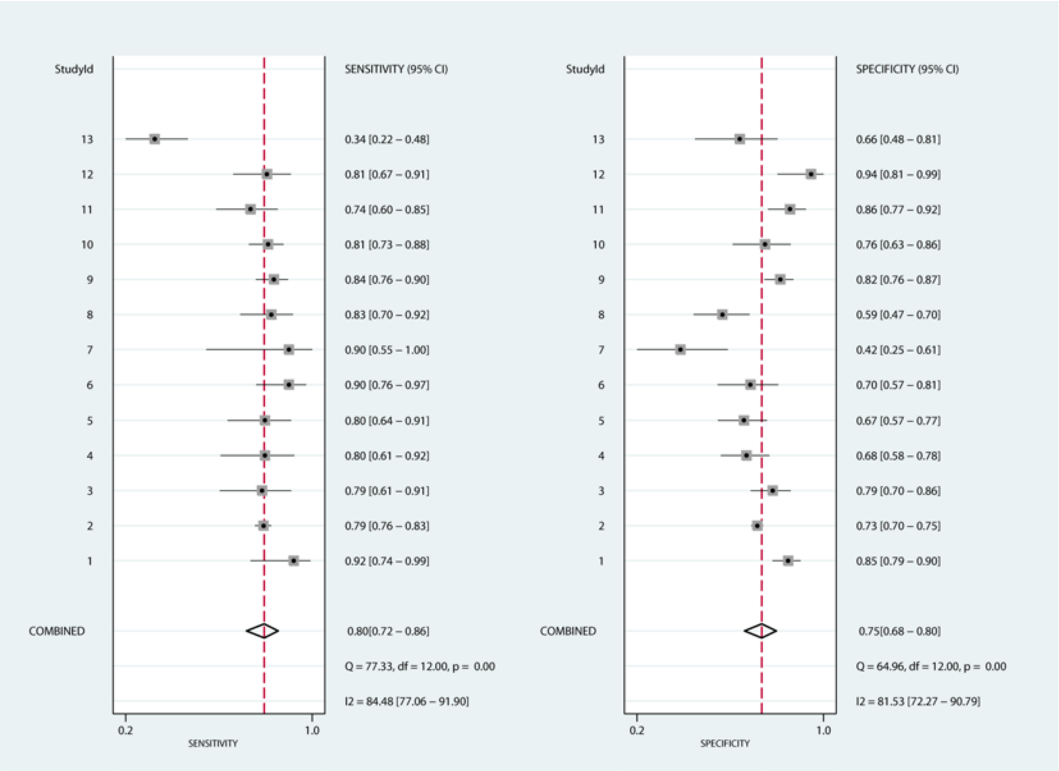

Figure S1. The summary of the results with regard to the sensitivity and specificity for CA 19-9

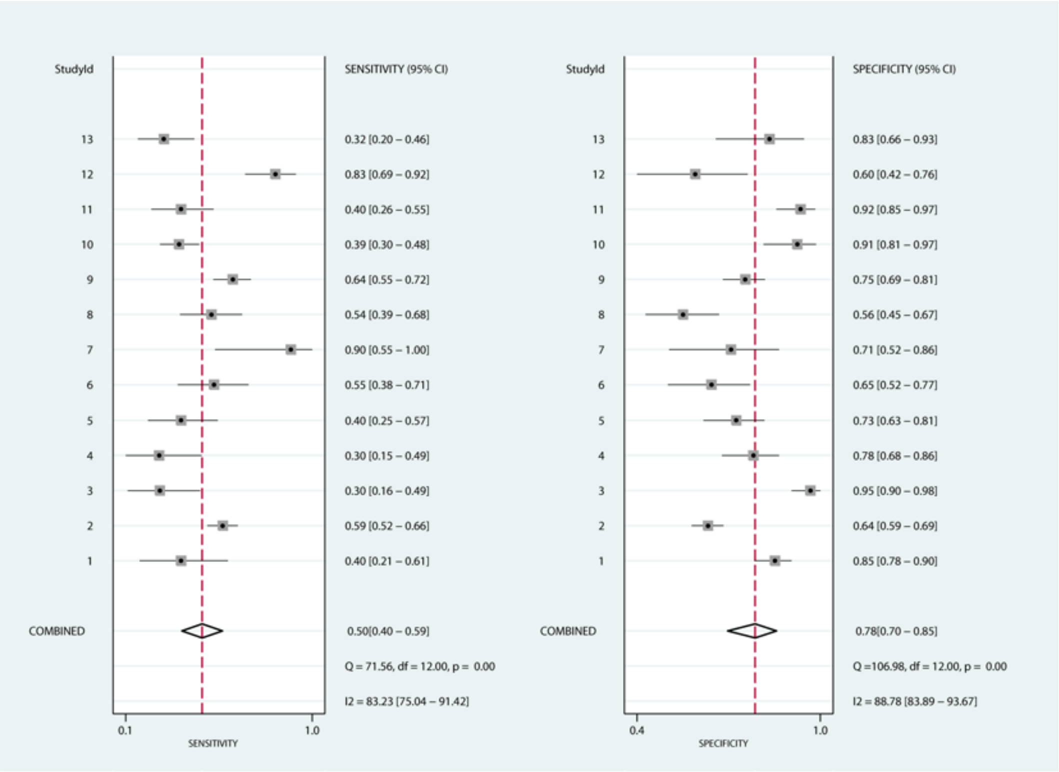

Figure S2. The summary of the results with regard to the sensitivity and specificity for CEA

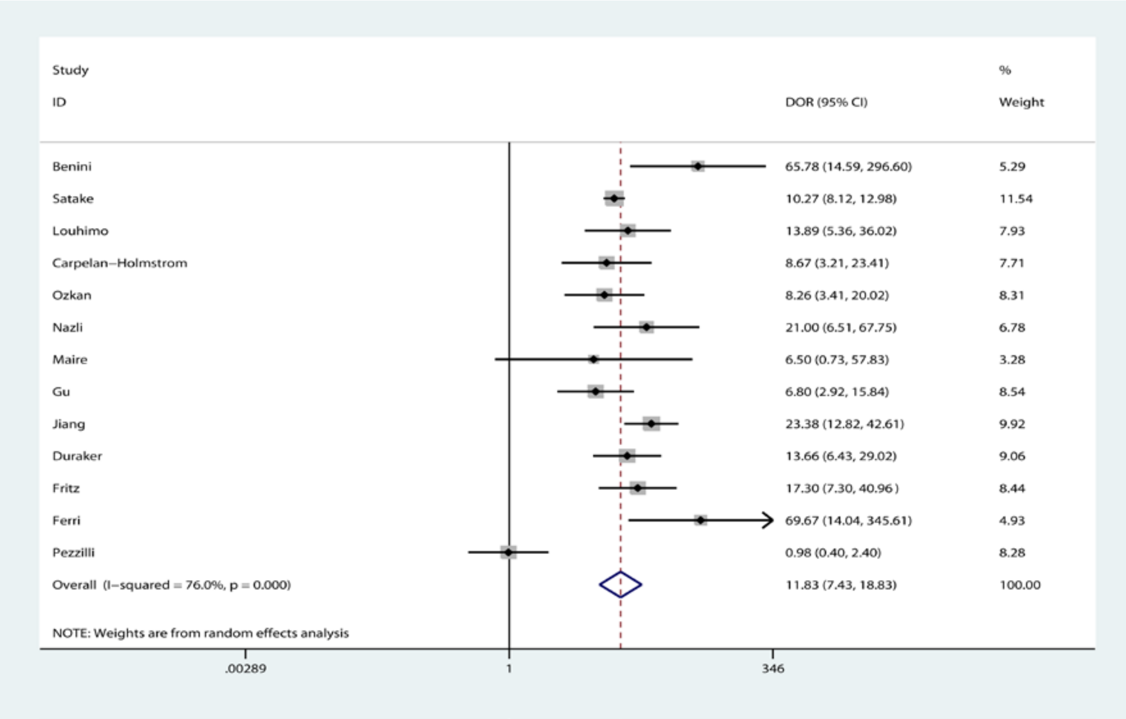

Figure S3. The summary of the results with regard to the DOR for CA 19-9

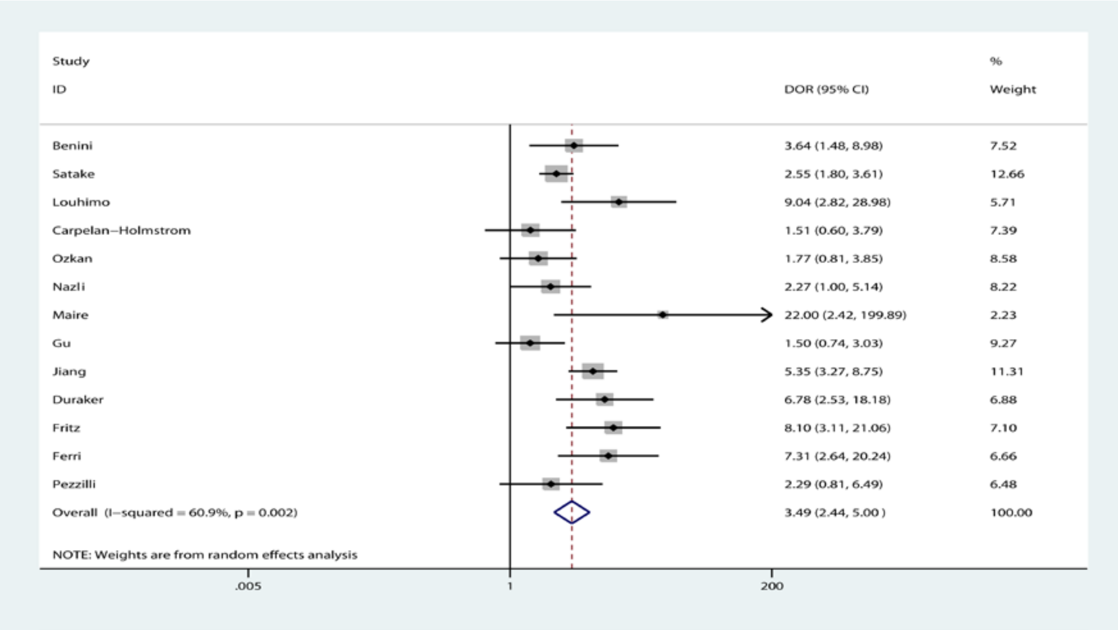

Figure S4. The summary of the results with regard to the DOR for CEA
